# A novel foveavirus identified in wild grapevine (*Vitis vinifera* subsp. *sylvestris*)

**DOI:** 10.1007/s00705-020-04817-x

**Published:** 2020-09-29

**Authors:** Jean-Sébastien Reynard, Justine Brodard, Eric Remoliff, Marie Lefebvre, Olivier Schumpp, Thierry Candresse

**Affiliations:** 1 Agroscope, route de Duiller 50, 1260 Nyon, Switzerland; 2 UMR 1332 BFP, INRA, Univ. Bordeaux, CS20032, 33882 Villenave d’Ornon cedex, France

## Abstract

We report the genome sequence of a putative new foveavirus infecting non-cultivated *Vitis vinifera*, tentatively named “grapevine foveavirus A” (GFVA). This virus was identified by high-throughput sequencing analysis of a European wild *Vitis* collected in Switzerland. Phylogenetic analysis revealed that this virus clustered with known grapevine virus T (GVT) isolates but was clearly distinct from any of them. If considering the International Committee of Taxonomy of Viruses (ICTV)-suggested foveavirus species demarcation criterion based on sequence similarity in the replicase gene/protein, this virus should be considered a member of a new species closely related to GVT. On the other hand, comparison of capsid gene/protein sequences using the same criteria indicates that GFVA is at the border of species demarcation. Whether this virus represents a highly divergent GVT isolate or a member of a distinct but closely related species is discussed.

**Electronic supplementary material:**

The online version of this article (10.1007/s00705-020-04817-x) contains supplementary material, which is available to authorized users.

Despite being an endangered taxon, wild European grapevine, *Vitis vinifera* subsp. *sylvestris* still occurs in very small isolated populations in forests or on rocky slopes in Switzerland [1]. Based on morphological criteria, seven individual vines of *Vitis vinifera* subsp. *sylvestris* were identified in three different locations in natural areas in Switzerland. During winter 2013, cuttings were collected and were vegetatively propagated and maintained in a greenhouse. The virome of these native wild grapevines was analyzed by high-throughput sequencing (HTS). Briefly, leaf petioles of the seven accessions were collected in summer 2017, and total RNA was extracted from each sample and subsequently pooled for library preparation without poly(A) purification (TrueSeq Stranded mRNA, Illumina). The library was sequenced on an Illumina HiSeq 4000 System (2 × 150 bp). A total of 118.3 million paired-end (PE) reads were obtained. The reads when then mapped to the genome of *Vitis vinifera* (PN40024 12X v2), and unmapped reads were assembled de novo using Geneious v11.1 (Biomatters Ltd). Finally, contigs were compared in a BLAST search (June 2020) to reference sequences for identification of viruses and viroids.

A single contig showing a distant relationship to the grapevine virus T (GVT) genome was identified and tentatively named “grapevine foveavirus A” (GFVA) (MN553040; length, 8,624 nt; number of mapped PE reads, 1475; mean coverage, 40.7), together with two other viruses: grapevine rupestris stem pitting-associated virus (GRSPaV) and grapevine rupestris vein feathering virus (GRVFV). No viroids were detected.

GVT is a member of the genus *Foveavirus* in the family *Betaflexiviridae*. It has recently been found by high-throughput sequencing in *Vitis vinifera* cv. Teroldego, a red Italian grape variety [4]. Since then, GVT has been reported in different countries, including Germany, France, Croatia, Slovakia, Italy, China and the Czech Republic, where the virus seems to be widespread [2, 7]. GRSPaV is another foveavirus infecting grapevine and is nearly ubiquitous among commercially cultivated grapevines worldwide [6].

The GFVA sequence covers most of the genome, extending to part of the 5′ and 3′ untranslated regions (48 and 169 nt in length, respectively). The genomic organization of GFVA is similar to that described for GVT and other foveaviruses. It has five open reading frames coding, respectively from 5′ to 3′, for the replicase (REP), the three triple-gene block proteins (TGB1-3, 221, 115 and 78 aa in length) and the coat protein (CP, 255 aa). The ORF1 product is two amino acids (aa) longer in GFVA (2131 aa) than in all known GVT isolates (2129 aa). The full genome nucleotide sequence identity of GFVA ranged from 68 to 70% as compared to GVT genome sequences available in the GenBank database. In phylogenetic analysis performed based on different genome regions (full genome, REP, CP), GFVA grouped together with known GVT isolates, forming a separate branch within a clade (Fig. 1).

**Fig. 1:**
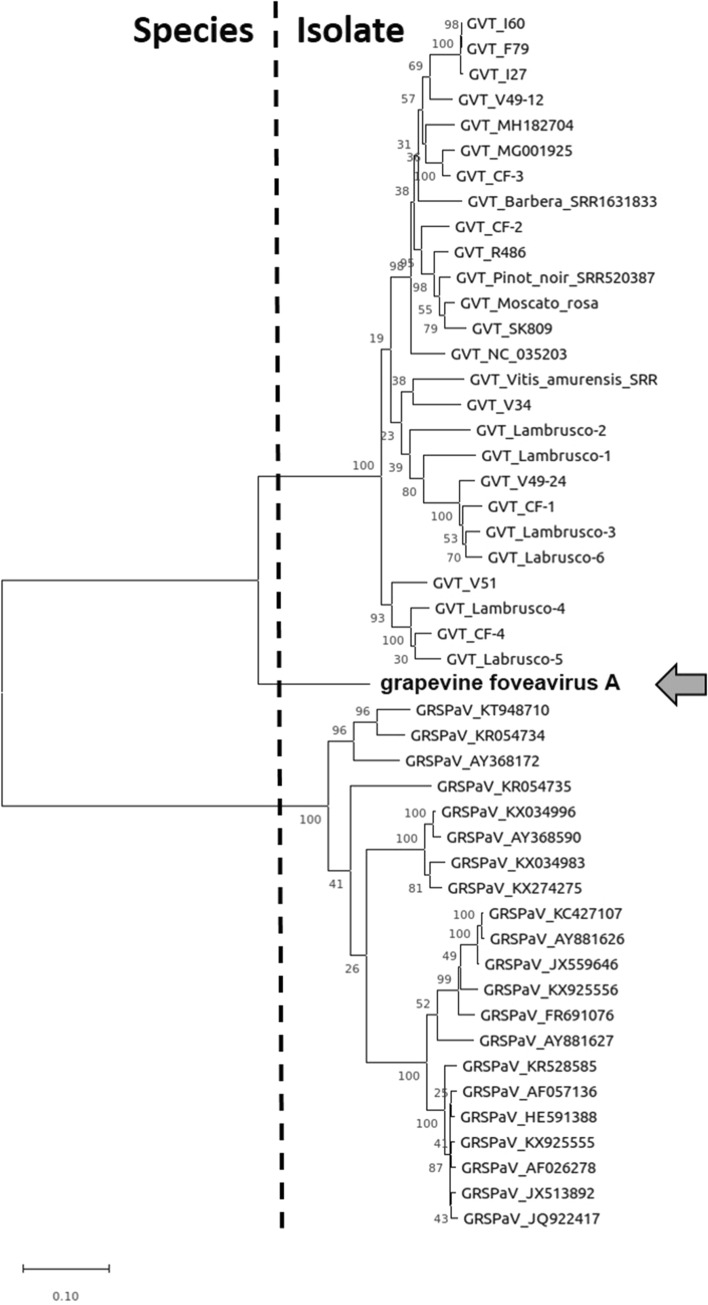
Phylogenetic tree constructed based on a multiple alignment of the complete amino acid sequence of the REP protein using ClustalW to indicate the relationship between grapevine foveavirus A (GFVA, arrow) and the other known grapevine-infecting foveaviruses. The scale represents 0.1 amino acid substitution per site. The alignment was done on a set including 26 grapevine virus T (GVT) and 21 grapevine rupestris stem pitting-associated virus (GRSPaV) isolates with completely sequenced genomes in order to represent the known diversity of both viruses [7]. The tree was generated by the maximum-likelihood algorithm with 500 bootstrap replicates using MEGAX. The cutoff of 80% aa sequence identity from ICTV for species demarcation in the genus *Foveavirus* is depicted on the tree

In pairwise comparisons, we observed that the genetic distance of GFVA from any GVT or GRSPaV isolate is higher than the known intraspecific diversity for both of those viruses (Table 1). The criteria of 80% amino acid (aa) sequence identity for the CP and REP proteins, or 72% nucleotide (nt) sequence identity for the corresponding genes have been suggested by the ICTV *Betaflexiviridae* Study Group for species demarcation in the genus *Foveavirus.* Using these criteria, contradictory conclusions can be reached depending on the gene/product considered. GFVA would represent a new species, sister to GVT, if considering the REP gene (nt sequence identity to GVT: 69–71%) or the REP protein (aa sequence identity to GVT: 73–77%). On the other hand, when examining the CP gene/protein, it should rather be regarded as a highly divergent isolate of GVT, although some CP protein comparisons fall below the species limit (nt sequence identity, 72–76%; aa sequence identity, 79–83%). Indeed, aa sequence identity values between GFVA CP and those of three of the 26 available GVT genome sequences are below the 80% identity species demarcation cutoff (Table 1). Therefore, when considering the CP gene/protein, differences between GFVA and GVT isolates are at the borderline of threshold values. In addition, CP protein identity between GFVA and known GVT isolates shows a clear discontinuity (Fig. S1). Considering REP/CP genes/proteins, the divergence of GFVA from GVT isolates is also clearly higher than what has been reported within the species when including isolates of GRSPaV, a grapevine virus characterized by a high genetic diversity [3]. There are already some situations in the family *Betaflexiviridae* in which the REP-based and CP-based species discrimination criteria have provided conflicting indications (*e.g.*, some viruses belonging to the genera *Vitivirus* and *Foveavirus*). The reverse situation to the one reported here, with the REP-based criteria suggesting a unique species and the CP-based ones suggesting distinct species, has also been observed, for example, in the case of isolates of Asian prunus viruses 1, 2 and 3 [5]. Such conundrums can only be solved by taking into consideration other species discrimination criteria, such as serology or other biological differences such as vector specificity or host range, but these are seldom available for newly described agents, in particular those infecting woody hosts. The alternative would be a revision of the sequence-based criteria for species demarcation, and it is noteworthy that the ICTV *Betaflexiviridae* study group is currently engaging in this discussion. In the specific case of GFVA, no additional information is available that could be used to reach a final decision about its taxonomic position, and therefore, this question should await the outcome of ongoing discussions on revision of species demarcation thresholds.

**Table 1:** Range of pairwise nucleotide and amino acid sequence identity values (%) for different isolates of grapevine virus T (GVT) and grapevine rupestris stem pitting-associated virus (GRSPaV)

	Full genome	REP (nt/aa)	TGB1 (aa)	TGB2 (aa)	TGB3 (aa)	CP (nt/aa)
GRSPaV isolates	76-99	74-99/83-89	85-99	78-100	76-100	81-99/90-99
GVT isolates	80-99	79-94/87-97	82-95	75-98	69-100	79-95/87-99
GFVA vs GVT	68-70	69-71/73-77	66-72	65-73	44-55	72-76/79-83
ICTV species demarcation		72/80				72/80

The alignment was done on 26 GVT isolates and 21 full-length GRSPaV genome sequences to be representative of the various phylogenetic groups identified for these two grapevine-infecting foveaviruses [7]

In order to confirm the HTS results, we tested the seven wild *Vitis* accessions separately by RT-PCR with GFVA-specific primers. Only one sample (accession St George 1) tested positive for GFVA. Furthermore, using flower sex genotyping [8], we determined that the GFVA-infected St George 1 accession is a male vine. This finding confirms that the St George 1 accession is a true *Vitis vinifera* subsp. *sylvestris*, since cultivated grapevines are hermaphrodite. No particular symptoms were observed on the St George 1 plant, indicating that GFVA might be latent. Whether infection by GFVA can be associated with minor effects on *Vitis vinifera* remains to be investigated.

## Electronic supplementary material

Below is the link to the electronic supplementary material.

Supplementary file1 (DOCX 63 kb)

## References

[1] Arnold C, Gillet J, Gobat JM (1998). Situation de la vigne sauvage Vitis vinifera ssp. silvestris en Europe. Vitis.

[2] Glasa M, Predajna L, Sihelska N, Soltys K, Ruiz-Garcia AB, Olmos A, Wetzel T, Sabanadzovic S (2018). Grapevine virus T is relatively widespread in Slovakia and Czech Republic and genetically diverse. Virus Genes.

[3] Hily JM, Beuve M, Vigne E, Demangeat G, Candresse T, Lemaire O (2018). A genome-wide diversity study of grapevine rupestris stem pitting-associated virus. Arch Virol.

[4] Jo Y, Song MK, Choi H, Park JS, Lee JW, Lian S, Lee BC, Cho WK (2017). Genome sequence of Grapevine Virus T, a novel foveavirus infecting Grapevine. Genome Announc.

[5] Marais A, Faure C, Candresse T (2016). New insights into Asian Prunus viruses in the light of NGS-based full genome sequencing. PLoS ONE.

[6] Meng B, Martelli GP, Golino D, Fuchs M (2017). Grapevine viruses: molecular biology, diagnostics and management.

[7] Nourinejhad Zarghani S, Hily JM, Glasa M, Marais A, Wetzel T, Faure C, Vigne E, Velt A, Lemaire O, Boursiquot JM, Okic A, Ruiz-Garcia AB, Olmos A, Lacombe T, Candresse T (2018). Grapevine virus T diversity as revealed by full-length genome sequences assembled from high-throughput sequence data. PLoS ONE.

[8] Fechter I, Hausmann L, Daum M, Rosleff Sörensen T, Viehöver P, Weisshaar B, Töpfer R (2012). Candidate genes within a 143 kb region of the flower sex locus in *Vitis*. Mol Genet Genomics.

