# Supplementary material for: A novel foveavirus identified in wild grapevine (Vitis vinifera subsp. sylvestris)
Source: Arch Virol. 2020 Sep 29;165(12):2999–3002. doi: 10.1007/s00705-020-04817-x (PMC7588375; doi:10.1007/s00705-020-04817-x)
Supplement: Supplementary file 1 — Supplementary file1 (DOCX 63 kb) [file 705_2020_4817_MOESM1_ESM.docx]

**Supplementary Materials**

**Figure S1** Distribution of the pairwise amino acid identity in coat protein (CP) sequences of different grapevine virus T (GVT) isolates and grapevine foveavirus A (GFVA). Twenty six GVT isolates were considered, as reported by Nourinejhad Zarghani *et al.* 2018. A total of 27 sequences were compared resulting in 351 different value for aa identity.

**
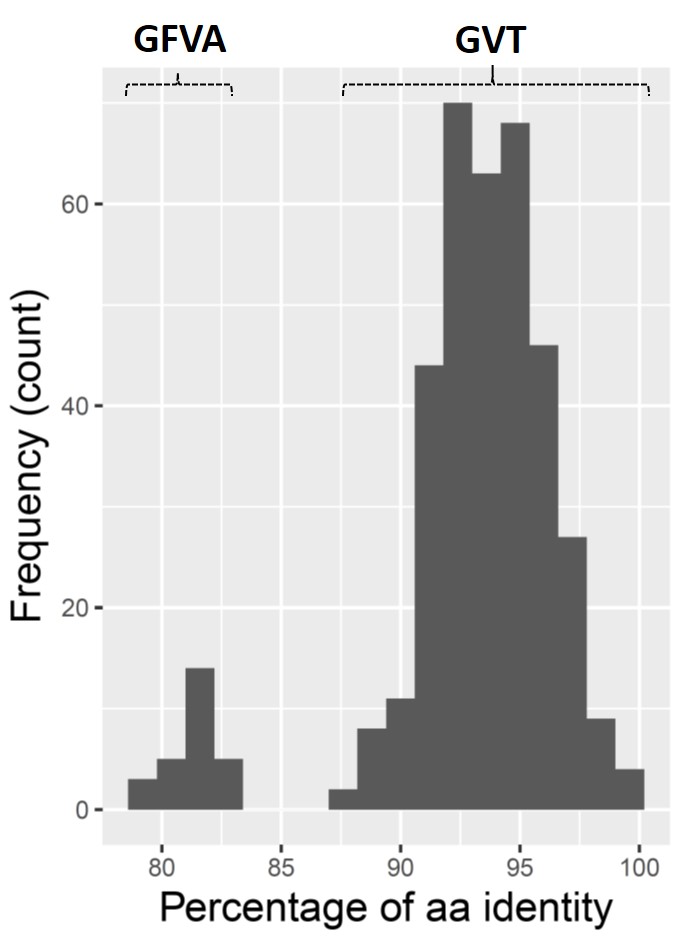
**
